# Multidrug Resistant *Mycobacterium tuberculosis*: A Retrospective *katG* and *rpoB* Mutation Profile Analysis in Isolates from a Reference Center in Brazil

**DOI:** 10.1371/journal.pone.0104100

**Published:** 2014-08-05

**Authors:** Flávia A. D. de Freitas, Vagner Bernardo, Michel K. Gomgnimbou, Christophe Sola, Hélio R. Siqueira, Márcia A. S. Pereira, Fátima C. O. Fandinho, Harrison M. Gomes, Marcelo E. I. Araújo, Philip N. Suffys, Elizabeth A. Marques, Rodolpho M. Albano

**Affiliations:** 1 Departamento de Bioquímica, Universidade do Estado do Rio de Janeiro, Rio de Janeiro, Brazil; 2 CNRS–Université Paris–Sud, Institut de Génétique et Microbiologie– Infection Genetics Emerging Pathogens Evolution Team, Orsay, France; 3 Faculdade de Ciências Médicas, Universidade do Estado do Rio de Janeiro, Rio de Janeiro, Brazil; 4 Centro de Referência Professor Hélio Fraga, Fundação Oswaldo Cruz, Rio de Janeiro, Brazil; 5 Laboratório de Biologia Molecular Aplicada a Micobacteria, Fundação Oswaldo Cruz, Rio de Janeiro, Brazil; University of Delhi, India

## Abstract

**Background:**

Multidrug resistance is a critical factor in tuberculosis control. To gain better understanding of multidrug resistant tuberculosis in Brazil, a retrospective study was performed to compare genotypic diversity and drug resistance associated mutations in *Mycobacterium tuberculosis* isolates from a national reference center.

**Methods and Findings:**

Ninety-nine multidrug resistant isolates from 12 Brazilian states were studied. Drug-resistance patterns were determined and the *rpoB* and *katG* genes were screened for mutations. Genotypic diversity was investigated by *IS6110*-RFLP and Luminex 47 spoligotyping. Mutations in *rpoB* and *katG* were seen in 91% and 93% of the isolates, respectively. Codon 315 *katG* mutations occurred in 82.8% of the isolates with a predominance of the Ser315Thr substitution. Twenty-five isolates were clustered in 11 groups with identical *IS6110*-RFLP patterns while 74 showed unique patterns with no association between mutation frequencies or susceptibility profiles. The most prevalent spoligotyping lineages were LAM (47%), T (17%) and Haarlen (12%). The Haarlen lineage showed a higher frequency of codon 516 *rpoB* mutations while codon 531 mutations prevailed in the other isolates.

**Conclusions:**

Our data suggest that there were no major multidrug resistant *M. tuberculosis* strains transmitted among patients referred to the reference center, indicating an independent acquisition of resistance. In addition, drug resistance associated mutation profiles were well established among the main spoligotyping lineages found in these Brazilian multidrug resistant isolates, providing useful data for patient management and treatment.

## Introduction

Tuberculosis (TB) is an infectious disease caused by bacteria of the *Mycobacterium tuberculosis* complex. In general, its clinical form is characterized by lung impairment, however, this disease may also affect other anatomical sites or occur in a disseminated form. TB is currently one of the main causes of morbidity and mortality affecting the main vulnerable groups (young adults, children and people with HIV) [1]. Multidrug resistant (MDR) *M. tuberculosis* strains are highly pathogenic and show great dissemination capacity. The global average of MDR-TB estimated for newly reported TB cases in 2012 was 3.6%, being slightly higher in high MDR-TB burden countries with 4.2% [1].

According to the World Health Organization (WHO), in 2012 there were an estimated 8.6 million new TB cases in the world representing a burden of 1.3 million deaths (including 320,000 deaths among HIV-positive patients). TB, therefore, is a highly relevant public health issue in Brazil, being the third cause of death from infectious diseases. Brazil is one of the 22 countries that account for 82% of all TB cases in the world [1]. In 2012, there were 82,000 new TB cases in this country with an incidence of 46/100,000 inhabitants and a mortality rate of 2.5/100,000. In 2012, of a total of 900 cases tested for MDR-TB in Brazil, 684 cases were laboratory confirmed as MDR.

The high TB prevalence is assumed to result from poverty, the HIV epidemic and inadequate diagnosis and treatment [2], [3]. Isoniazid (INH) and rifampin (RMP) are still the most important drugs available for TB treatment. Multidrug resistance (MDR), defined as resistance to at least RMP and INH, is another critical factor involved in TB control as MDR *M. tuberculosis* strains are highly pathogenic and have the potential for great dissemination. While the standard treatment requires six months of chemotherapy the regimen for most MDR-TB patients takes 20 months. In 2012, an estimated 450,000 people developed MDR-TB worldwide with 170,000 deaths [1]. Most cases in 2012 were in South-East Asian (29%), African (27%) and Western Pacific (19%) regions [1].

Global data collected in 2012 from MDR surveys and continuous surveillance among notified TB cases suggest that 3.6% of newly diagnosed TB cases and 20% of those previously treated for TB showed MDR-TB. The highest levels of MDR-TB are found in Eastern Europe and Central Asia where in some countries more than 20% of new TB cases and more than 50% of those previously treated for TB are MDR. In 2012, of a total of 94,000 TB patients eligible for MDR-TB treatment, 84,000 cases were confirmed. This represented a 42% increase in MDR cases compared with 2011 [1].

Gene mutations are typically associated with resistance to specific drugs in *M. tuberculosis*. RMP has been the most important drug available for TB treatment. It inhibits gene transcription by binding to the beta subunit of the DNA dependent RNA polymerase, encoded by the *rpoB* gene. Most *rpoB* mutations occur in a 81 bp region known as RRDR (RMP resistance determining region), located between codons 507 and 533 [4].

Several genes can be involved in INH resistance (*katG*, *inhA*, *KasA*, *ndh*, the *oxyR*-*ahpC* intergenic region, *fabG*, *fadE24*, *inhA* promoter, *iniA* and the *mabA-inhA* operon) [5], [6]. The *katG* gene encodes the catalase-peroxidase enzyme that is involved in the activation of the pro-drug INH. The loss or reduction of enzyme activity by mutations prevents this process, allowing survival in the presence of INH [7], [8]. *katG* gene mutations are present in most INH resistant *M. tuberculosis* clinical isolates where alterations in codon 315 predominate [9], [10]. Mutations located at the *mabA-inhA* operon can also lead to INH resistance and are present in 8–30% of the resistant strains [6]. Mutations in the other genes are rare and usually coexist with other hotspot mutations in *katG* and *mabA-inhA* [6].

Molecular techniques that differentiate *M. tuberculosis* strains are important tools in public health issues such as TB outbreaks and to unravel transmission patterns. The RFLP (restriction fragment length polymorphism) analysis of the IS*6110* insertion element (IS*6110*-RFLP), when performed under standardized conditions, is highly reproducible and effective in transmission studies in a specific region or place or between populations, providing better ways to understand and control disease transmission [11], [12].

The basis for spoligotyping (spacer oligonucleotide typing) lies in the determination of the direct repeat number present in a specific region of the *M. tuberculosis* genome [13]. Alone or in conjunction with other techniques, spoligotyping can be used to establish strain/isolate relationships, investigate circulating strains, transmission dynamics and the natural history of TB [14]–[18].

Here we present a retrospective analysis of *katG* and *rpoB* mutation profiles and the genotypic diversity by IS*6110*-RFLP and spoligotyping of 99 MDR *M. tuberculosis* isolates obtained at a MDR-TB national reference center in Rio de Janeiro. Our goals were to determine correlations between genotypes and *katG* and *rpoB* mutations and evaluate relationships within these isolates and the main global circulating *M. tuberculosis* strains. This information could be useful for TB care and control as Brazil has a considerable incidence of primary resistance which is, typically, 6% and 1.5% for INH and RMP, respectively [19].

## Materials and Methods

### Mycobacterium tuberculosis isolates

Ninety-nine MDR *M. tuberculosis* isolates were provided by the National Tuberculosis Reference Center for Multidrug Resistance Prof. Hélio Fraga, in Rio de Janeiro, Brazil. According to international standards, MDR is defined as resistance to at least INH and RMP. These *M. tuberculosis* isolates were obtained from patients with pulmonary TB who were referred to the Center between 1995 and 2003. Fifty-three isolates were from patients that resided in Rio de Janeiro State and 46 isolates were from patients living in 12 other Brazilian States (Amazonas: 3; Pará: 5; Maranhão: 4; Ceará: 2; Paraíba: 3; Pernambuco: 5; Bahia: 2; Goiás: 3; Minas Gerais: 4; São Paulo: 6; Paraná: 7 and Rio Grande do Sul: 2) (Figure S1). Isolates were selected according to two different criteria: one group (20 isolates) belonged to a National Survey on Drug Resistant Tuberculosis performed in Brazil between 1995 and 1997. This survey was undertaken by the Brazilian TB control program to detect the magnitude of drug resistance in TB treatment. Unfortunately, clinical data collection was not prioritized in the survey so it was available only for few patients. Among the 5,586 patients with pulmonary TB enrolled in the National Survey, 2% (123 patients) were MDR. Clinical data was available for only one of these patients therefore the other 19 isolates from this MDR group were randomly selected. The remaining 79 isolates were from MDR-TB patients referred to the Reference Center Professor Helio Fraga for treatment. Patietnts were referred to the center by State Clinics who characterized resistance to TB treatment. We tried to select patients for whom clinical data were available and who were admitted for treatment between 2002 and 2003 to match the last years of the survey. Clinical data was available for 36 of these patients due to the destruction of patient records during a flood in the Center. Strain isolation was achieved from sputum samples by cultivation in *Löwenstein*-*Jensen* (LJ) medium.

### Drug susceptibility tests

Drug resistance patterns were determined on LJ medium using the proportions method at critical concentrations and critical proportions of the population indicating bacterial resistance. The concentrations and proportions were 40 mg/L and 1.0% for RMP, 0.2 mg/L and 1.0% for INH, 100 mg/L and 10% for pirazinamid (PZA), 4 mg/L and 10% for streptomycin (St) and 2.0 mg/L and 1.0% for ethambutol (EMB) [20].

### DNA extraction

*M. tuberculosis* isolates were cultured for three to four weeks at 37°C in LJ medium. Genomic DNA was prepared by a CTAB protocol as described by van Soolingen *et al.* (1991) [21]. Genomic DNA was inspected by agarose gel electrophoresis and quantified on a spectrophotometer (Nanoview, GE Healthcare, Brazil).

### Genotyping by IS6110-RFLP

DNA fingerprinting by IS*6110* RFLP was performed on the isolates following standard procedures [22]. Briefly, extracted mycobacterial DNA was digested with *PvuII*, separated by electrophoresis, transferred to a Hybond N^+^ membrane and hybridized with a horseradish peroxidase conjugated 245-bp PCR-amplified probe directed against the right arm of IS*6110*. Detection was performed with the ECL Direct Labeling and Detection System (GE Healthcare Life Sciences, Brazil). Autoradiograms were scanned at 190 dots/inch and IS*6110* patterns were analyzed using Bionumerics (Windows version 4.0, Applied Maths). The position of IS*6110* containing fragments was normalized against the internal markers and the accuracy of the procedure was verified by comparison of IS*6110* banding patterns to those of strain Mt.14323 on different autoradiograms. Similarity matrices were constructed using the Dice band-based similarity coefficient using a band tolerance interval of 2.0% of the pattern length and dendrograms were generated by the hierarchic unweighted pair group method analysis (UPGMA) clustering algorithm.

### Genotyping by Luminex spoligotyping

Spoligotyping was performed as described previously [23]. We used the Luminex 200 platform and spoligotype patterns were entered in the SITVIT2 proprietary database of the Institut Pasteur de la Guadeloupe, which is an updated version of the previously released SpolDB4 database (available online http://www.pasteur-guadeloupe.fr:8081/SITVITDemo) [24]. Some samples were classified as new because they did not match any pattern of the Spoligotype International Type (SIT) that designates patterns shared by two or more patient isolates. Therefore, a new spoligotype pattern is one that has never been reported before. Some isolates were also classified as unknown, which means that they do have an SIT number but the lineage to which they belong is unknown.

### *katG* and *rpoB* mutation analysis

Mutation analysis of the *katG* gene (GenBank accession number U06258) was concentrated on two regions of interest. The first, extending from the first codon (GTG/valine) to codon 119, was PCR amplified using primers *katG1* sense, 5′-A CTTCGCGATCACATCCGTG-3′ and *katG1* antisense,5′-GCGGCCGTCGTGGATGCGGTA-3′. The second region, from codon 267 to 504, was amplified with primers *katG2* sense, 5′-CGGCGGTCACACTTTCGGTA-3′ and *katG2* antisense 5′-CCCGACTTGTGGCTGCAGGC-3′. The region comprehending codons 445 to 560 of the *rpoB* gene (GenBank accession number L27989) was PCR amplified with primers *rpoB* sense 5′-CATCGACCACTTCGGCAACCG-3′ and *rpoB* antisense 5′-TTTCGATCGGGCACATCCGGC-3′. All PCR reactions were performed using Platinum *Taq* High Fidelity (Invitrogen, Brazil), 1.5 mM of MgCl_2_, 15 pmol of each primer, 0.2 mM of dNTPs, 1X PCR buffer (provided by the manufacturer) and 40 ng of genomic DNA. PCR parameters were as follows: denaturation at 95°C for 5 min, followed by 35 cycles at 95°C for 45 s, 56°C (for *rpoB*) and 63°C (for *katG*) for 45 s and 72°C for 1 min, and a final extension time of 10 min at 72°C. Amplicons were analyzed by agarose gel electrophoresis, purified by the Illustra GFX PCR DNA and Gel Band Purification kit (GE Healthcare, Brazil), sequenced directly with the DYEnamic ET terminator cycle sequencing kit (GE Healthcare) and analyzed on a MegaBACE 1000 automated DNA sequencer platform (GE Healthcare). Several reactions in both directions were done for each amplicon to ensure accurate sequencing. Chromatograms were analyzed using Chromas software (version 2.22, Technelysium Pty Ltd, Australia). The consensus sequences of each sample were compared to the wild-type *M. tuberculosis katG* and *rpoB* gene sequences in order to identify the mutations. Blast searches against Genbank were performed for nucleotide comparisons.

### MabA-15 MAS-PCR

MabA-15 MAS-PCR was carried out as described previously [6].

### Statistical analysis

Data was analyzed with GraphPad Prism 5.0 software (GraphPad Software Inc. La Jolla, CA, USA). Associations between categorical variables were assessed using the Chi-square test or Fisher's exact test. A *P* value of <0.05 was considered statistically significant. Ninety-five percent confidence intervals of all proportions were calculated by Wald's equation: 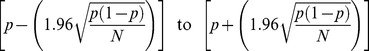



Where: 
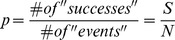



## Results and Discussion

As described above, clinical data was available for only 37 patients and is shown in Table S1. Among them, 15 were women and 22 were men. Globally, most TB cases and deaths occur in men but this disease remains among the top three killers of women [1]. The median age at the diagnosis of MDR was 33 years. The main type of resistance was acquired resistance (89%; CI = 82.7–95.1) and almost 50% of the patients died after two years of follow up.

All of the 99 MDR isolates analyzed were resistant to INH and RMP, 52 were resistant to EMB and 35 to streptomycin ST (Table 1). Drug susceptibility results for some of the isolates could not be determined due to intrinsic difficulties of the method. Susceptibility patterns for all samples are shown in Table S2.

**Table 1:** Resistance patterns of the 99 MDR isolates analyzed.

Resistance pattern				n
INH	RMP	EMB	ST	
R	R	S	S	32
R	R	R	R	25
R	R	R	S	23
R	R	R	ND	4
R	R	ND	ND	3
R	R	S	R	8
R	R	ND	R	2
R	R	S	ND	2

R- Resistant;

S- Sensitive;

ND-Not determined.

Mutations in the 81-bp RRDR region of the *rpoB* gene were present in 90 isolates. A total of 51, 23 and 11 isolates carried mutations at codons 531, 526 and 516, respectively, confirming this segment of the gene as a hotspot region, as observed worldwide [10], [25]–[32]. The other five isolates had mutations distributed among other codons (Table 2). Furthermore, eight isolates that had mutations in codon 531, 526 or 516, also had mutations in other codons. Therefore, 82 isolates exhibited single changes but seven presented double mutations, always in conjunction with the altered codons 531, 526 or 516. One isolate had point mutations in three separate codons (531, 522 and 539).

**Table 2:** Mutation patterns for the *rpoB* and *katG* genes found in 99 MDR isolates.

Gene	Mutation			n
	Mutated codon	Specific Mutation	Amino acid change	
*rpoB*	531	TCG - TTG	Ser - Leu	44
		TCG - TGG	Ser - Trp	7
	513	CAA - CCA	Gln - Pro	2
	516	GAC - GTC	Asp - Val	3
		GAC - TAC	Asp - Tyr	8
	526	CAC - GAC	His - Asp	9
		CAC - TAC	His - Tyr	8
		CAC - CGC	His - Arg	2
		CAC - TGC	His - Cys	2
		CAC - AAC	His - Asn	2
	511	CTG - CCG	Leu - Pro	4
	475*	GTG - GGG	Val - Gly	1
	522*	TCG - TTC	Ser - Phe	1
	539*	TCA - TTC	Ser - Phe	1
	533	CTG - CCG	Leu - Pro	1
	545	CTG - CCG	Leu - Pro	2
	508*	ACC - CCC	Thr - Pro	1
	471*	ATG - ATT	Met - Ile	1
*katG1*	4	A deletion at position 60		26
	65*	G deletion at position 241		2
	17*	AGC - ACC	Ser - Thr	1
	92–93*	T insertion at position 325		1
	2*	C deletion at positon 54		1
	11*	C deletion at positon 81		1
	26*	G deletion at positon 126		1
	107	G deletion at positon 368		1
	67	G deletion at positon 249		1
	115*	T isertion at position 392		1
	1*	GTG - GCG	Val - Ala	1
	93	GCC- ACC	Ala - Thr	1
*katG2*	315	AGC - ACC	Ser - Thr	75
		AGC - AAC	Ser - Asn	3
		AGC - ACA	Ser - Thr	3
		AGC - ATC	Ser - Ile	1
	463	CGG - CTG	Arg -Leu	2
	431	G deletion at positon 1293		1
	399*	GAA - GAG	Glu - Glu	2
	493*	A deletion at positon 1525		1
	439	G insertion at position 1365		1
	485*	G deletion at positon 1501		1

*associated with another mutated codon.

We found four new alleles for the *rpoB* gene in codons 475, 539, 545 and 471. Three of them occurred as double mutations involving always one of the most frequently mutated codons. Three of these alleles were from isolates from Rio de Janeiro state while the one in codon 471 was from a Pernambuco isolate. It is possible that these double mutations are related to compensatory mutations. Several studies have demonstrated that drug resistance mutations in essential genes may have a negative impact on key physiological functions. Drug resistance incurs a fitness cost and compensatory mutations in target or related genes can help to reduce it [33].

We previously reported *katG* mutation profiles for 64 of the isolates included in this study [34] and now the remaining 35 samples were analyzed. Two regions of this gene were sequenced, *katG1* and *katG2*, the latter containing codon 315, the most commonly mutated one [10], [25], [26], [35]–[38]. *katG* mutations were found in 92 isolates with seven of them showing mutations only in *katG1*. Among these 92 isolates, 82 had changes in codon 315 and 5 showed double mutations in *katG2* in codons 463, 399, 493 or 485 that were always accompanied by codon 315 alterations and are probably compensatory mutations (Table 2). Although amino acid changes in codon 431 have already been described [39], we also found a new single nucleotide (G) deletion in this codon. Within *katG1*, 30 isolates showed mutations in which 26 were deletions in codon 4. The remaining 4 isolates showed insertions, deletions and base substitutions in this region (Table 2). The full results of the mutation screening for the *rpoB* and *katG* genes can be seen in Table S3.

Three MDR isolates had no *katG* and *rpoB* mutations and 6 others were RMP resistant but also had no *rpoB* mutations. Curiously, although most samples in this study were from Rio de Janeiro state, only one of these 6 isolates was from this state. Additionally, 4 other isolates were INH resistant but had no *katG* mutations. It is possible that some of these isolates possess an efflux mechanism of drug resistance, extruding structurally and functionally unrelated compounds by transporter pumps. This mechanism can be responsible for intrinsic and acquired drug resistance in prokaryotic and eukaryotic cells [40].

Moreover, especially regarding resistance to INH, mutations in other genes can be involved. We also investigated through multiplex allele-specific polymerase chain reaction (MAS-PCR), mutations in the *mabA*-15 region in the 8 INH resistant isolates that carried the wild type *katG* gene and found that only one had a mutation in this region (data not shown).

This investigation not only confirms data observed in other studies analyzing the resistance and mutation profiles of Brazilian MDR isolates but also describes new mutations in isolates that were collected over an eight-year period from different Brazilian states. Furthermore, the mutation profiles described in our data could help in the design of rapid diagnostic tests that simultaneously detect TB and MDR by new strategies such as high resolution melting analysis in qPCR.

To evaluate whether mutation frequencies could be influenced by clustered isolates, their possible genetic relationships were investigated through IS*6110*–RFLP (Table 3). The number of IS*6110* copies detected ranged from 2 to 18 with an average number of 10. Twenty-five isolates were clustered in 11 groups comprising 2 or 3 isolates with identical RFLP patterns. The remaining 74 isolates showed unique IS*6110* patterns and there was no association between mutation frequencies at each mutated codon or susceptibility profiles.

**Table 3:** IS*6110*-RFLP fingerprint clusters and *rpoB* and *katG* mutations for clustered samples.

Cluster	Isolate code	Resistance profile	*rpoB* mutation	*katG* mutation
1	2316/02	INH, RMP	Ser531Leu	Ser315Thr, Del 4
	2410/02PA*	INH, RMP, EMB, ST	His526Cys	Ser315Thr
2	235/03	INH, RMP,EMB	Ser531Trp	Ser315Thr, Del 4
	243/03	INH, RMP, EMB	Ser531Trp	Ser315Thr
3	274/03	INH, RMP, EMB	Ser531Leu	Ser315Thr
	CE02	INH, RMP, ST	NM	Ser315Thr
	MG 285	INH, RMP	NM	Ser315Thr
4	009/03	INH, RMP, EMB, ST	Ser531Trp	Ser315Thr
	720/03	INH, RMP, EMB, ST	Ser531Trp	Ser315Thr
5	2475/02	INH, RMP, EMB, ST	Ser531Leu	Ser315Thr
	682/03	INH, RMP	Ser531Leu	Ser315Thr
6	1275/03	INH, RMP, ST	Asp516Tyr	Ser315Asn
	2010/97	INH, RMP, EMB, ST	Gln513Pro	Ser315Thr
	865/03	INH, RMP	Ser531Leu	Ser315Thr/Del 485
7	1194/03	INH, RMP, EMB, ST	His526Asp	Ser315Thr
	529/03MA	INH, RMP	His526Tyr	Ser315Thr, Del 4
8	847/03	INH, RMP, EMB, ST	Leu511Pro	Ser315Thr
			Asp516Tyr	
	850/03	INH, RMP, EMB, ST	Asp516Tyr	Ser315Thr, Del 4
9	1010/03	INH, RMP, EMB	Ser531Leu	Ser315Thr
	PE76	INH, RMP	His526Tyr	Ser315Thr
	PA107	INH, RMP, ST	NM	Ser315Thr
10	1221/03	INH, RMP	Ser531Leu	Ser315Thr
	480/03	INH, RMP	His526Asn	Ser315Thr, Del 4
11	1269/03	INH, RMP	Ser531Leu	Ser315Thr
	276/03	INH, RMP	Ser531Leu	Ser315Thr, Del 4

NM- No mutations in the studied region.

*The Brazilian state from where the isolate was obtained is indicated as PA-Pará state; AM-Amazonas state; MA-Maranhão state. All the other isolates came from Rio de Janeiro state.

The susceptibility tests for pyrazinamide (PZA) were also conducted but these results were mostly inconclusive and therefore omitted.

Two RFLP clusters (cluster 4 and cluster 5) with 2 isolates each showed similar resistance profiles and carried single *rpoB* and *katG* mutations at codon 531 and 315, respectively. However, in cluster 5 the resistance profile was different for each isolate concerning EMB and ST. The transmission of the same MDR isolate between the patients of these 2 clusters may have happened, as suggested by the fact that all isolates in these clusters came from Rio de Janeiro state and showed similar drug resistance patters and identical *rpoB* and *katG* mutations.

Regarding the other nine clusters, no apparent mutation or resistance profile relationships were detected. Their *rpoB* and *katG* mutations were somewhat different with some showing distinct amino acid changes (clusters 6 and 7) or different mutations at the same gene (*rpoB* mutations in clusters 1, 3, 6, 8, 9, 10). Interestingly, in several clusters a *katG* codon 4 deletion was found in one of the isolates belonging to the same cluster (clusters 1, 2, 6, 7, 8, 10, 11) (Table 3). This indicates that transmission of the same isolate happened before the MDR phenotype was established, suggesting an independent acquisition of antibiotic resistance. Moreover, clusters 1, 3, 7 and 9 exhibited isolates that were from different states, indicating that the same strain may have circulated freely between different Brazilian regions. Seven clusters showed different mutation profiles for the *rpoB* or *katG* gene. These data suggest that there were no major MDR strains transmitted between patients in Brazil between the years 1995 and 2003.

We observed a high rate of *IS6110-*RFLP polymorphisms among the 99 MDR isolates that is again indicative of independent acquisition of resistance. This is not surprising since secondary resistance was the main mode of MDR development in our samples. The opposite was observed for MDR isolates from Rio Grande do Sul by Perizzolo *et al.* (2012) [30], who had a large level of genotype clustering and concluded that these came mostly from strains that had been transmitted in the last few years.

**Figure 1:**
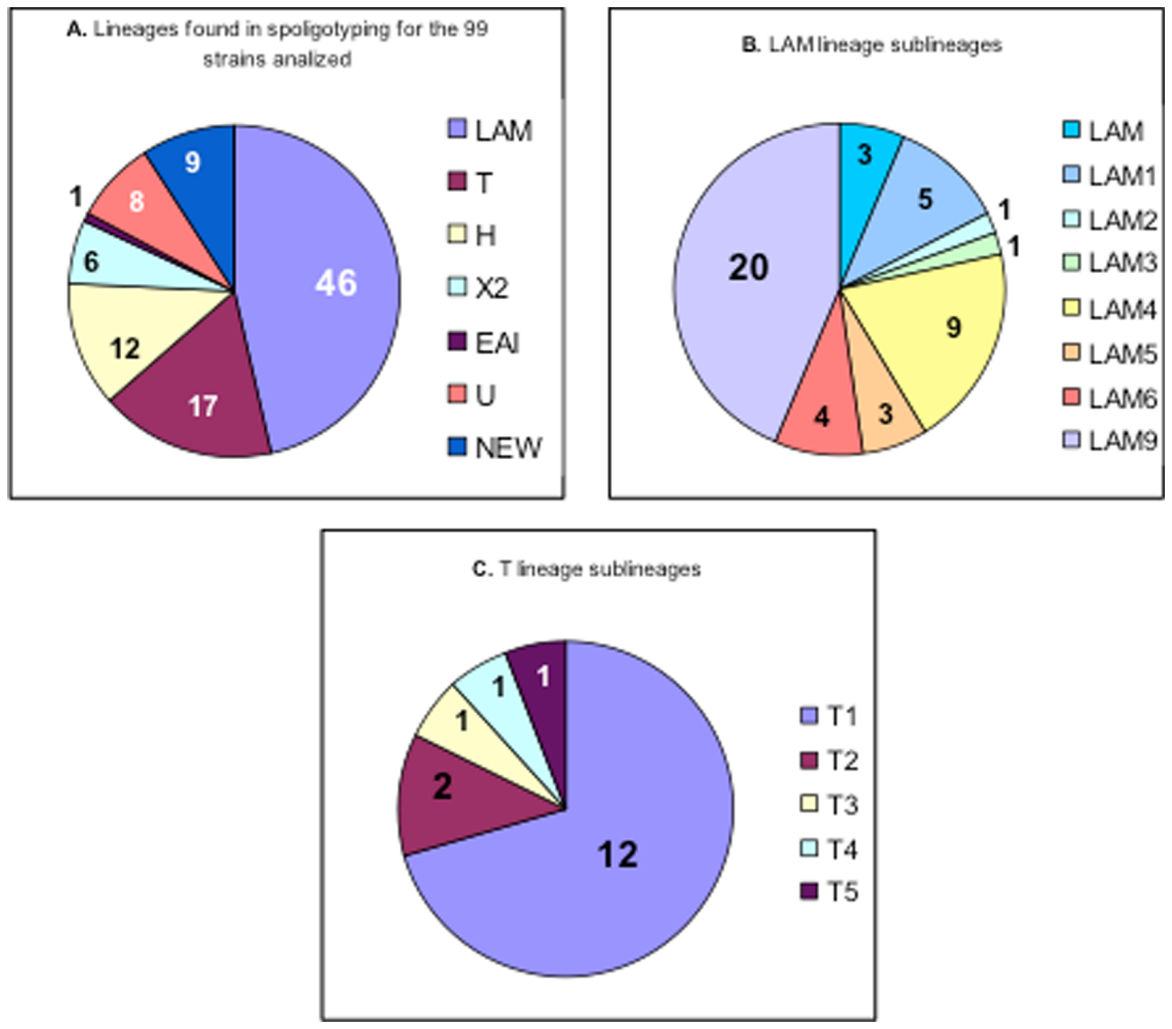
Spoligotyping analysis of MDR-TB isolates. Eighty-two isolates were classified into 5 distinct lineages (Figure 1A). Isolates of LAM Family were distributed into 8 sublineages (Figure 1B) and Isolates of T Family were distributed into 5 sublineages (Figure 1C). U – Unknown, LAM - Latin-America and Mediterranean, T - T super-family, H - Haarlen, EAI - East African Indian.

Spoligotyping was also performed for all samples (Figure 1A). An updated version of the SpolDB4 database, the SITVIT2 proprietary database of the Institut Pasteur de la Guadeloupe, was used to identify all spoligotype patterns. Eighty-two isolates were classified into 5 distinct lineages (Figure 1A). Forty-six were assigned to the Latin-America and Mediterranean (LAM) family, 17 to T super-family 12 to Haarlen (H), 6 to X2 and 1 to the East African Indian (EAI) families. Recently, a large spoligotyping analysis reported that LAM, T and H lineages are the most frequently observed ones in Brazilian *M. tuberculosis* isolates [31]. Our study corroborates with these findings as we found very similar frequencies for these lineages in our MDR isolates.

Of the other 17 isolates, 9 were classified as new and 8 as unknown. No statistically significant differences in lineage frequencies were found between samples from Rio de Janeiro and those from other states (Chi-square test, p = 0.43). This indicates that our data is general enough and could be useful for the development of diagnostic tests for MDR-TB in Brazil (Table 4). Lineage distribution among the 37 patients for whom clinical data was available was not statistically different from the whole set (Chi-square test, p = 0.77)

**Table 4:** Demographic data and TB incidence in different Brazilian regions.

Brazilian regions	States	Population^a^	TB incidence^b^	Number (%)of isolates in study
North	Amazonas	3,221,939	66.71	3 (3.0%)
	Pará	7,065,573	45.78	5 (5.0%)
Northeast	Ceará	8,185,286	41.99	2 (2.0%)
	Pernambuco	8,485,386	47.81	5 (5.0%)
	Maranhão	6,118,995	39.73	4 (4.0%)
	Paraíba	3,641,395	27.56	3 (3.0%)
	Bahia	14,080,654	41.25	2 (2.0%)
Central west	Goiás	5,647,035	14.59	3 (3.0%)
Southeast	Minas Gerais	19,273,506	23.99	4 (3.0%)
	Rio de Janeiro	15,420,375	73.27	53 (53.53%)
	São Paulo	39,827,570	36.51	6 (6.0%)
South	Paraná	10,284,503	24.70	7 (7.0%)
	Rio Grande do Sul	10,582,840	41.51	2 (2.0%)

a From: http://saladeimprensa.ibge.gov.br/en/noticias?view=noticia&id=1&busca=1&idnoticia=1065 (Instituto Brasileiro de Geografia e Estatística-IBGE, population in 2007).

b From: http://tabnet.datasus.gov.br/cgi/deftohtm.exe?idb2009/d0202.def (Indicadores e Dados Básicos-IDB, Tb incidence in 2007).

Incidence rate: new cases/100,000 inhabitants.

Isolates of LAM and T families were distributed into 8 and 5 sublineages, respectively (Figure 1B and 1C). In LAM family, 20 isolates were assigned to LAM9 and 9 to LAM4 sublineages while for the T family, T1 was the major sublineage with 12 isolates. Haarlem family isolates were also subdivided into H1 and H3 sublineages with 2 and 10 isolates, respectively (data not shown).

*IS6110*-RFLP and spoligotyping patterns, octal number, SIT and lineage for each isolate can be observed in Table S4. Curiously, the EAI lineage, that shows a phylogeographic distribution in South-East Asia, India and East-Africa, was represented by one isolate from Parana state. This raises the question of whether transmission from other continents may have happened.

IS*6110*–RFLP clusters were compared with spoligotyping data (Table 5). As expected, isolates belonging to the same RFLP cluster were of similar spoligotype lineages. Our data allowed us, through a paired analysis, to discriminate some *IS6110*-RFLP clusters by spoligotyping. Some isolates within the same *IS6110*-RFLP and spoligotyping cluster had different mutation profiles and came from various Brazilian states. This reinforces the idea of independent acquisition of drug resistance in these clusters. We can also note that for RFLP clusters 10 and 11, isolates were assigned as new profiles in LAM sublineage. Both isolates in cluster 11 showed identical RFLP patterns and octal numbers and were tentatively identified as LAM3 sublineage members.

**Table 5:**
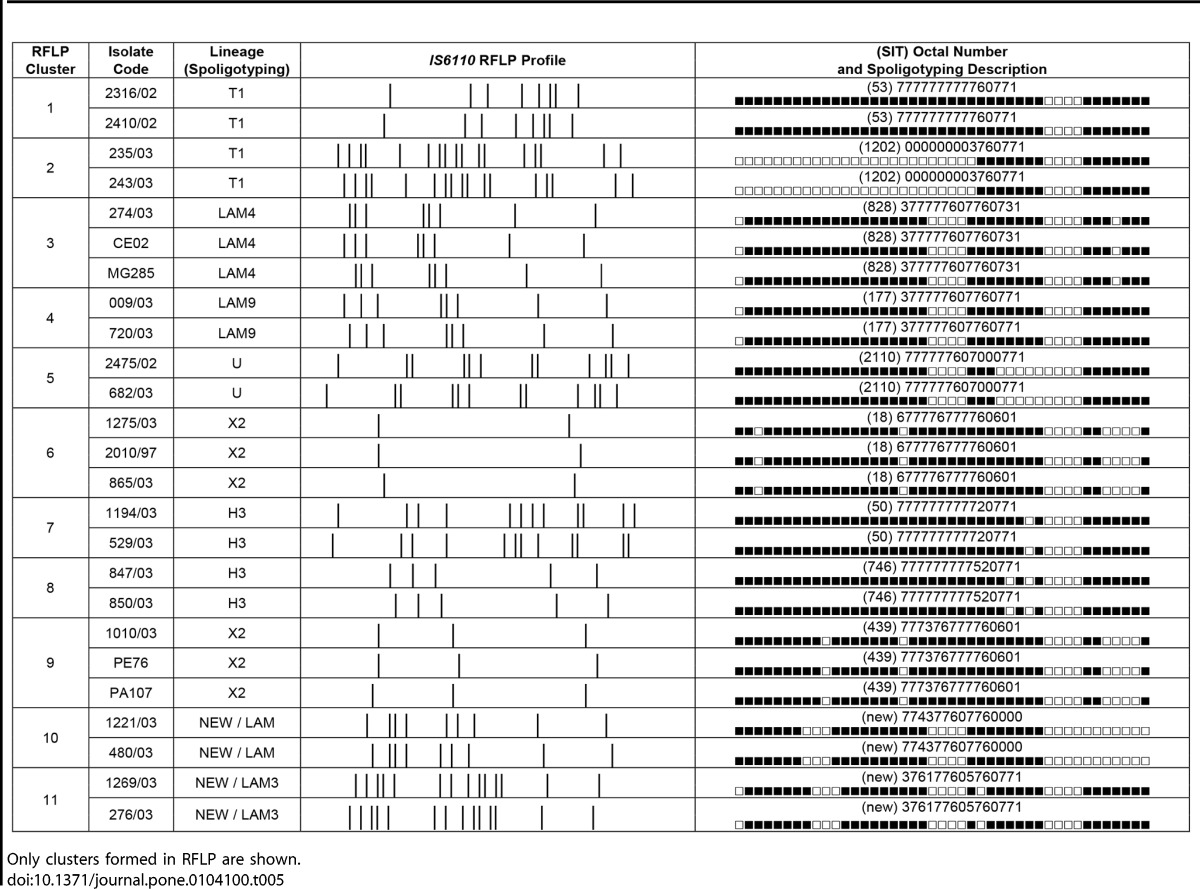
*IS6110*-RFLP cluster patterns and their corresponding shared types.

Only clusters formed in RFLP are shown.

A relationship, although not statistically significant (Fischer test, p = 0.17), between the absence of *katG* codon 4 deletions with lineage X2 was observed (Table 6). Although codon 4 deletions were common in isolates from all lineages, X2 was the only clustered lineage that did not have them. More isolates belonging to this lineage should be analyzed to try to reach statistical significance and confirm this hypothesis. However, we suggest that in countries where this lineage is common, MDR patient care should take into account that these patients may carry this mutation. Considering the H lineage, *rpoB* gene mutations were atypical, with codon 516 being the most frequently altered followed by 526 and 531 (Chi-square test, p<0.0001). However, for the other lineages the most commonly mutated codons were those normally seen in RMP resistant isolates worldwide (codons 531, 526 and 516).

**Table 6:** Association between spoligotyping lineages and *rpoB* and *katG* mutations in 99 MDR strains.

Characteristic	Number (%)* of isolates					
	Spoligotype lineage					Total
	LAM	T	H	X2	EAI 1	
A.*rpoB* mutation						
531	27 (58.7%)	11 (57.9%)	1 (7.7%)	2 (33.3%)	0 (0%)	41
526	11 (23.9%)	2 (10.5%)	4(30.8%)	1 (16.7%)	1 (25%)	19
516	1 (2,2%)	2 (10.5%)	6(46.1%)	1 (16.7%)	0 (0%)	10
Other mutations	6 (13%)	2 (10.5%)	2 (15.4%)	1 (16.7%)	0 (0%)	11
B.*katG* mutation						
315	37 (80.4%)	13 (72.2%)	9 (75.0%)	6 (100%)	1 (100%)	66
Del 4	11 (23.9%)	6 (33.3%)	4 (30.8%)	0 (0%)	1 (100%)	22
Other mutations	14 (30.4%)	3 (15.8%)	1 (7.7%)	1 (16.7%)	0 (0%)	20
Total (A+B)	107	39	27	12	3	189

*The percentage of mutations refers to the total number of mutations in each isolate. The Unknown and New types were excluded from this table.

A comparative analysis of our data with a study that analyzed 217 MDR *M. tuberculosis* isolates from Russia in a similar way showed that 46%, 10% and 2% of them belonged to the LAM, H and T lineages, respectively [32]. While only 4 T lineage isolates were observed in the Russian samples, we had 17 (17.1%; CI = 9.7–24.6). Another major difference was seen for *rpoB* codon 531 that was the most frequently mutated one in our study while no mutations were found in the Russian samples. Russian LAM isolates showed *rpoB* mutations in codon 516 (75%) and 531 (7%) and also in *katG* at codon 315 (99%). Similarly, 46.4% (CI = 36.6–56.3) of our MDR isolates also belonged to LAM lineage with a high frequency of codon 315 mutations (82.6%; CI = 71.7–93.6). However, we observed a much higher frequency (60.9%; CI = 46.8–75) of codon 531 *rpoB* mutations, with only 2% (CI = 0–6.4) in codon 516.

In H lineage Russian isolates, 43% had no *katG* mutations against 16.7% (CI = 0–37.8) in our study. For *rpoB*, mutations were absent in 48% of them against only 8.3% (CI = 0–24) in ours. Additionally, mutations in *rpoB* codon 516 were the most frequent ones in our H lineage isolates while the same was not observed for the Russian samples. However, our results are similar to another study in Brazil with 121 MDR isolates from Rio Grande do Sul state [30], in which 20% had no *katG* and 12% no rpoB mutations. These observations suggest that the frequencies of certain mutations in particular genotypic lineages are dependent on the geographical region of the isolates.

Perizzolo *et al*. (2012) [30] also observed that only 4% of their samples from Rio Grande do Sul state belonged to the H lineage while we had 12% (CI = 5.7–18.6) among all samples, which suggests that this difference may be occurring only when Rio Grande do Sul state is analyzed separately from other Brazilian states. They also observed that the largest sublineage was LAM5 (15%), one that is rarely observed among drug susceptible samples in this region. In our study, the frequency of the LAM5 sublineage was low (3%; CI = 0–6.4), which is similar to what was found in drug susceptible samples from Rio Grande do Sul and reinforces the idea that Perizzolo *et al*. (2012) [30] analyzed samples from a specific MDR outbreak. Concerning the type of mutations found in LAM5, both studies showed similar findings, with one isolate that had no mutations and several with mutations in codons 531 (*rpoB*) and 315 (*katG*). The mutation profile in this sublineage seems to be very similar in Brazilian isolates.

The two main spoligotyping lineages found in our study, LAM and T, were compared with their respective mutation profiles (Table 7). There were some associations between *katG1* alterations and the sublineages. LAM6 and LAM9 carry a higher number of *katG1* deletions than the other LAM sublineages (Chi-square test, p = 0,008). Furthermore, all isolates belonging to lineages LAM and T also had mutations in *katG2* codon 315. Another interesting observation, although not statistically significant, is that the T lineage shows much lower mutation diversity in both *rpoB* (Chi-square test, p = 0.50) and *katG* (Chi-square test, p = 1.00) than LAM.

**Table 7:** The main spoligotyping lineages with all associated mutation profiles.

Clade	Number of isolates	*rpoB* mutations	*katG1* mutations*	*katG2* mutations*
LAM	3	531 (2)*, 526 (1)	NM	315 (2)
LAM 1	5	531 (3), 526 (2), 511 (1)	Del 4 (1)	315 (5)
LAM 2	1	531 (1)	Del 4 (1)	315 e 399 (1)
LAM 3	1	531 (1)	17, ins 92–93 (1)	315 (1)
LAM 4	9	531 (3), 526 (4), 508 (1)	NM	315 (9), 493 (1)
LAM 5	3	531 (2)	NM	315 (2)
LAM 6	4	531 (4)	Del 2 (1), Del 4 (3), Del 11 (1), Del 65 (1)	315 (1), Del 431 (1)
LAM 9	20	531 (11), 526 (4), 516 (1), 522 (1), 539 (1), 545 (1), 533 (1)	Del 4 (6), Del 26(1), Del 65 (1), Del 67 (1), Del 107 (1)	315 (16), 399 (1), 463 (1)
T 1	12	531 (8), 526 (2), 516 (1), 513 (1)	Del 4 (3)	315 (9), 439 (1)
T 2	2	531 (2)	Del 4 (1), ins 115 (1)	315 (1)
T 3	1	516 (1)	Del 4 (1)	315 (1)
T 4	1	511 (1)	Del 4 (1)	315 e 463 (1)
T 5	1	531 (1)	NM	315 (1)

NM - No mutation.

*katG1 mutations and *katG2* mutations refer to mutations in the first and in the second regions of the gene analyzed, respectively.

*The numbers in brackets mean the number of times that each mutation appears.

MDR-TB is one of the most important threats for TB control. Sensitivity tests of first line drugs strongly influence patient care and the utilization of resources before the treatment. In this work, a correlation of drug resistance profiles with *IS6110* fingerprinting clusters and *rpoB* and *katG* mutations was attempted in order to determine possible associations. We found that isolates belonging to the same cluster did not necessarily show the same resistance or mutation profile, indicating that genotyping to strain level cannot predict resistance in this set of samples. This type of investigation could be optimized by a new Luminex based method, the Spoligoriftyping that allows the simultaneous assessment of the main *rpoB* mutation SNPs (codons 516, 526 and 531), the main mutations in genes related to INH resistance (*katG* 315, *inhA* −15) and spoligotyping [41].

Although most isolates came from Rio de Janeiro, we also analyzed isolates from other 11 Brazilian states, as shown in Table 4. Thus, we were able to draw an idea of *rpoB* and *katG* mutations throughout the Brazilian territory. Even though alterations in at least three genes (*katG*, *inhA* and *ahpC*) are associated with INH resistance, our analysis, as confirmed in other studies, shows that *katG* mutations are good predictors of resistance as 93% (CI = 87.9–98) of our INH resistant isolates carried mutations in this gene. Among these, 89% (CI = 82.8–95.5) had codon 315 mutations with a predominance of the Ser315Thr substitution. This profile is consistent with findings from other countries [25], [26], [36], [37], [38] and also with previous studies of Brazilian MDR isolates [10], [39]. In spite of that, one study with INH resistant Brazilian isolates from Goiás state [42] found a lower prevalence of codon 315 *katG* mutations (6 of 17), raising the question of whether geographical strain differences could be responsible for this variation.

According to Costa *et al.* (2009) [43], there is an association between the *katG* 315 mutation and the H lineage in strains from South America (Brazil, Peru and Argentina) but there is none with the LAM lineage. In contrast, we had a high number of codon 315 mutations in these two lineages. Although the Ser315Thr mutation results in an enzyme incapable of activating INH, it retains approximately 50% of the catalase-peroxidase activity [44]. Therefore, this altered enzyme provides high-level resistance to INH while still keeping a level of oxidative protection that creates a clear advantage for the isolates that carry it.

In the present study, we correlated data from resistance and mutation profiles with spoligotyping and *IS6110*-RFLP cluster analysis. Such data are highly relevant for the study of global TB transmission. One study with 99 MDR isolates from Portugal, for example, showed that *IS6110*-RFLP in association with *rpoB* mutation analysis can be useful to unambiguously confirm isolate identities and to propose epidemiological links [45]. Another study with 114 isolates recovered from patients of a Russian prison [46] determined the active transmission of MDR isolates between prisoners through the high rate of MDR and the identical RFLP patterns, spoligotypes and *rpoB* mutations among new cases. The high proportion of clustered isolates (79.8%) in this prison clearly indicates an active and recent disease transmission.

Regarding the 37 isolates from patients who had clinical data available, no significant associations between spoligotype lineages and the following variables were found: sex, presence of comorbidities, patient outcome, presence of cavitation and pulmonary bilateral involvement (Chi-square test, p always>0.05).

In spite of being a high TB and HIV burden country, there is scarce data comparing *rpoB* and *katG* gene mutations with *IS6110*-RFLP and spoligotype fingerprints in MDR isolates in Brazil. Our data are useful to enhance our understanding of the mechanisms of resistance and their relationship with specific lineages. Furthermore, resistance profiles were well established in the present study among the main lineages found in Brazilian isolates (LAM and T), thus providing useful data for patient management and treatment in this country. Additionally, the characterization of these MDR isolates could aid in the development of diagnostic tools as well as guide new strategies to fight MDR-TB in Brazil.

## Supporting Information

Table S1 Clinical data available for a subset of the patients.
(DOCX)

Table S2 Table with the susceptibility patterns for the 99 MDR isolates analyzed.
(DOCX)

Table S3 Table with the mutational profiles of 99 multidrug resistant isolates.
(DOCX)

Table S4 Table with cluster associations between *IS6110* - RFLP and Spoligotyping data.
(DOC)

Figure S1 Map of Brazil showing the different states and the number of isolates analyzed.
(PDF)
