# Supplementary material for: Multidrug Resistant Mycobacterium tuberculosis: A Retrospective katG and rpoB Mutation Profile Analysis in Isolates from a Reference Center in Brazil
Source: PLoS One. 2014 Aug 5;9(8):e104100. doi: 10.1371/journal.pone.0104100 (PMC4122415; doi:10.1371/journal.pone.0104100)
Supplement: Table S1 — Clinical data available for a subset of the patients. (DOCX) [file pone.0104100.s001.docx]

| Table S1. Patients´ clinical characteristics (n=37) | | | |
| --- | --- | --- | --- |
| **Variable** | **Category** | **No. of cases** | **% of cases** |
| Gender |  |  |  |
|  | Male | 22 | 59.5 |
|  | Female | 15 | 40.5 |
| Comorbidities* |  |  |  |
|  | Yes | 9 | 24.4 |
|  | No | 28 | 75.6 |
| Type of resistance |  |  |  |
|  | Acquired | 33 | 89.1 |
|  | Primary | 2 | 5.45 |
|  | Unknown | 2 | 5.45 |
| Pulmonary involvement |  |  |  |
|  | Unilateral | 6 | 16.3 |
|  | Bilateral | 31 | 83.7 |
| Cavitation |  |  |  |
|  | Yes | 27 | 72.9 |
|  | No | 10 | 27.1 |
| Outcome (two years follow up) |  |  |  |
|  | Remission | 11 | 29.7 |
|  | Progression | 7 | 18.9 |
|  | Death | 18 | 48.6 |
|  | Unknown | 1 | 2.8 |
| Lineage |  |  |  |
|  | T | 6 | 16.3 |
|  | LAM | 15 | 40.5 |
|  | H | 7 | 18.9 |
|  | Other | 9 | 24.3 |
|  | | | |
| Age at MR diagnosis | | (median; 25%percentile - 75%percentile) | |
|  | | 33 years; 26 – 43 years | |

* (AIDS – 2 patients; alcoholism – 4 patients; asthma- 1 patient; mental disorder – 1 patient, Chronic obstructive pulmonary disease - 1 patient).
