# Supplementary material for: Multidrug Resistant Mycobacterium tuberculosis: A Retrospective katG and rpoB Mutation Profile Analysis in Isolates from a Reference Center in Brazil
Source: PLoS One. 2014 Aug 5;9(8):e104100. doi: 10.1371/journal.pone.0104100 (PMC4122415; doi:10.1371/journal.pone.0104100)
Supplement: Table S2 — Table with the susceptibility patterns for the 99 MDR isolates analyzed. (DOCX) [file pone.0104100.s002.docx]

**Table S2:** Susceptibility patterns for the 99 MDR isolates analyzed.

| Isolate code | Resistance pattern | | | | |
| --- | --- | --- | --- | --- | --- |
|  | INH | | RMP | EMB | ST |
|  |  |  |  |  |  |
| 111 | R | | R | S | S |
| 009 | R | | R | R | R |
| 039 | R | | R | S | S |
| 1010 | R | | R | R | S |
| 1085 | R | | R | R | -- |
| 1126 | R | | R | R | -- |
| 1194 | R | | R | R | R |
| 1205 | R | | R | -- | -- |
| 1221 | R | | R | S | S |
| 1251 | R | | R | S | S |
| 1264 | R | | R | R | S |
| 1269 | R | | R | S | S |
| 1275 | R | | R | S | R |
| 1314 | R | | R | R | S |
| 1316 | R | | R | R | R |
| 1411 | R | | R | R | S |
| 156 | R | | R | -- | R |
| 1576 | R | | R | S | S |
| 1740 | R | | R | R | R |
| 2010/02 | R | | R | R | R |
| 2010/97 | R | | R | R | R |
| 2056 | R | | R | R | R |
| 2153 | R | | R | R | R |
| 2197 | R | | R | S | R |
| 229 | R | | R | S | R |
| 2316 | R | | R | S | S |
| 232 | R | | R | R | -- |
| 235 | R | | R | R | S |
| 2355 | R | | R | R | R |
| 2410 | R | | R | R | R |
| 2414 | R | | R | R | S |
| 2417 | R | | R | S | S |
| 243 | R | | R | R | S |
| 247 | R | | R | R | R |
| 2475 | R | | R | R | R |
| 2505 | R | | R | R | S |
| 262 | R | | R | R | R |
| 263 | R | | R | R | R |
| 265 | R | | R | R | S |
| 2668 | R | | R | R | S |
| 2678 | R | | R | R | S |
| 2688 | R | | R | R | S |
| Continuation of the Table S1 | | | | | |
| 2689 | | R | R | R | R |
| 2697 | | R | R | S | S |
| 270 | | R | R | S | S |
| 274 | | R | R | R | S |
| 276 | | R | R | S | S |
| 2788 | | R | R | R | R |
| 2791 | | R | R | R | S |
| 294 | | R | R | S | R |
| 310 | | R | R | R | R |
| 314 | | R | R | R | S |
| 319 | | R | R | S | S |
| 32 | | R | R | R | S |
| 379 | | R | R | S | S |
| 415 | | R | R | R | R |
| 433 | | R | R | R | S |
| 480 | | R | R | S | S |
| 491 | | R | R | S | R |
| 529 | | R | R | S | S |
| 543 | | R | R | S | S |
| 563 | | R | R | R | S |
| 605 | | R | R | -- | -- |
| 640 | | R | R | S | -- |
| 682 | | R | R | S | S |
| 711 | | R | R | S | S |
| 720 | | R | R | R | R |
| 78 | | R | R | R | R |
| 790 | | R | R | S | S |
| 826 | | R | R | R | -- |
| 836 | | R | R | S | S |
| 837 | | R | R | R | R |
| 847 | | R | R | R | R |
| 850 | | R | R | R | R |
| 865 | | R | R | S | -- |
| 884 | | R | R | R | S |
| 885 | | R | R | R | R |
| 886 | | R | R | R | S |
| 89 | | R | R | -- | -- |
| 933 | | R | R | R | S |
| 96 | | R | R | -- | R |
| 972 | | R | R | R | S |
| 976 | | R | R | R | S |
| 981 | | R | R | R | R |
| 452 | | R | R | S | S |
| 358 | | R | R | S | S |
| 378 | | R | R | S | S |
| Continuation of the table S1 | | | | | |
| 02 | | R | R | S | R |
| 155 | | R | R | S | S |
| 150 | | R | R | S | S |
| 231 | | R | R | S | S |
| 285 | | R | R | S | S |
| 107 | | R | R | S | R |
| 03 | | R | R | S | S |
| 07 | | R | R | S | S |
| 11 | | R | R | S | S |
| 76 | | R | R | S | S |
| 261 | | R | R | S | R |
| 88 | | R | R | S | S |

R – Resistant

S – Sensitive

ND – Not determined
