# Supplementary material for: Multidrug Resistant Mycobacterium tuberculosis: A Retrospective katG and rpoB Mutation Profile Analysis in Isolates from a Reference Center in Brazil
Source: PLoS One. 2014 Aug 5;9(8):e104100. doi: 10.1371/journal.pone.0104100 (PMC4122415; doi:10.1371/journal.pone.0104100)
Supplement: Table S3 — Table with the mutational profiles of 99 multidrug resistant isolates. (DOCX) [file pone.0104100.s003.docx]

**Table S3:** Mutational profiles of 99 multidrug resistant isolates.

| Isolate code | Gene | | | | | |
| --- | --- | --- | --- | --- | --- | --- |
|  | *rpoB* | | *KatG1* | | *KatG2* | |
|  | Mutated codon | Codon/amino acid change(s) | Mutated codon | Codon/amino acid change(s) | Mutated codon | Codon/amino acid change(s) |
| 111 | 513 | Gln CAA - Pro CCA | -- | none | 315 | Ser AGC - Thr ACC |
| 09 | 531 | Ser TCG - Trp TGG | -- | none | 315 | Ser AGC - Thr ACC |
| 39 | 516 | Asp GAC - Val GTC | -- | none | -- | none |
| 1010 | 531 | Ser TCG - Leu TTG | -- | none | 315 | Ser AGC - Thr ACC |
| 1085 | 516 | Asp GAC - Tyr TAC | -- | none | 315 | Ser AGC - Thr ACC |
| 1126 | 531 | Ser TCG - Leu TTG | -- | none | 315 | Ser AGC - Thr ACC |
| 1194 | 526 | His CAC - Asp GAC | -- | none | 315 | Ser AGC - Thr ACC |
| 1205 | 531 | Ser TCG - Leu TTG | -- | none | 315 | Ser AGC - Thr ACC |
|  | 475 | Val GTG - Gly GGG |  |  |  |  |
| 1221 | 531 | Ser TCG - Leu TTG | -- | none | 315 | Ser AGC - Thr ACC |
| 1251 | -- | none | -- | none | 315 | Ser AGC - Thr ACC |
| 1264 | 531 | Ser TCG - Leu TTG | -- | none | 315 | Ser AGC - Thr ACC |
| 1269 | 531 | Ser TCG - Leu TTG | -- | none | 315 | Ser AGC - Thr ACC |
| 1275 | 516 | Asp GAC - Tyr TAC | -- | none | 315 | Ser AGC - ASN AAC |
| 1314 | -- | none | -- | none | -- | none |
| 1316 | 531 | Ser TCG - Leu TTG | 4 + 65 | 4 (Del A) + 65 (Del G) | -- | none |
| 1411 | 531 | Ser TCG - Leu TTG | -- | none | 315 | Ser AGC - Thr ACC |
| 156 | 531 | Ser TCG - Leu TTG | 17 + 92-93 | 17 (Ser AGC - Thr ACC) + 92-93 (Ins T) | 315 | Ser AGC - ACA Thr (2 nucleotides) |
| 1576 | 531 | Ser TCG - Leu TTG | -- | none | 315 | Ser AGC - Thr ACC |
| 1740 | 531 | Ser TCG - Leu TTG | 2 + 4 + 11 | 2 (Del C) + 4 (Del A) + 11 (Del C) | 431 | Del (G) |
| 2010/02 | 531 | Ser TCG - Leu TTG | 4 | 4 (Del A) | -- | none |
| 2010/97 | 513 | Gln CAA - Pro CCA | -- | none | 315 | Ser AGC - Thr ACC |
| 2056 | 526 | His CAC - Asp GAC | 4 | 4 (Del A) | 315 | Ser AGC - Thr ACC |
| **Table S2:** Mutational profiles of 99 multidrug resistant isolates. (*Continued*) | | | | | | |
| 2153 | 531 | Ser TCG - Leu TTG | -- | none | 315 | Ser AGC - Thr ACC |
|  | 522 | Ser TCG - Phe TTC |  |  |  |  |
|  | 539 | Ser TCA - Phe TTC |  |  |  |  |
| 2197 | 526 | His CAC - Tyr TAC | 4 + 26 + 65 | 4 (Del A) + 26 (Del G) + 65 (Del G) | 315 | Ser AGC - Thr ACC |
| 229 | 531 | Ser TCG - Leu TTG | 107 | 107 (Del G) | -- | none |
| 2316 | 531 | Ser TCG - Leu TTG | 4 | 4 (Del A) | 315 | Ser AGC - Thr ACC |
| 232 | 531 | Ser TCG - Trp TGG | 4 | 4 (Del A) | 315 | Ser AGC - Thr ACC |
| 235 | 531 | Ser TCG - Trp TGG | 4 | 4 (Del A) | 315 | Ser AGC - Thr ACC |
| 2355 | 526 | His CAC - Arg CGC | 4 | 4 (Del A) | 315 | Ser AGC - Thr ACC |
| 2410 | 526 | His CAC - Cys TGC | -- | none | 315 | Ser AGC - Thr ACC |
| 2414 | 511 | Leu CTG - Pro CCG | 4 | 4 (Del A) | 315 + 463 | 315 (Ser AGC - Thr ACC) + 463 (CGG Arg - CTG Leu) |
| 2417 | 531 | Ser TCG - Leu TTG | -- | none | 315 | Ser AGC - Thr ACC |
| 243 | 531 | Ser TCG - Trp TGG | -- | none | 315 | Ser AGC - Thr ACC |
| 247 | 516 | Asp GAC - Tyr TAC | -- | none | 315 | Ser AGC - Thr ACC |
| 2475 | 531 | Ser TCG - Leu TTG | -- | none | 315 | Ser AGC - Thr ACC |
| 2505 | 531 | Ser TCG - Leu TTG | -- | none | 315 | Ser AGC - Thr ACA (2 nucleotides) |
| 262 | 526 | His CAC - Tyr TAC | 4 | 4 (Del A) | 315 | Ser AGC - Thr ACC |
| 263 | 531 | Ser TCG - Leu TTG | -- | none | 315 + 399 | 315 (Ser AGC - Thr ACC) + 399 (Glu GAA - Glu GAG) |
| 265 | 531 | Ser TCG - Leu TTG | -- | none | 315 + 493 | 315 (Ser AGC - Thr ACC) + 493 (Del A) |
| 2668 | 531 | Ser TCG - Leu TTG | 4 | 4 (Del A) | 315 | Ser AGC - Thr ACC |
| 2678 | 531 | Ser TCG - Leu TTG | 67 | 67 (Del G) | -- | none |
| 2688 | 516 | Asp GAC - Val GTC | 4 | 4 (Del A) | 315 | Ser AGC - Thr ACC |
| 2689 | 526 | His CAC - Asp GAC | -- | none | 315 | Ser AGC - Thr ACC |
| 2697 | -- | none | -- | none | -- | none |
| 270 | 531 | Ser TCG - Leu TTG | -- | none | 315 | Ser AGC - Thr ACC |
| 274 | 531 | Ser TCG - Leu TTG | -- | none | 315 | Ser AGC - Thr ACC |
| 276 | 531 | Ser TCG - Leu TTG | 4 | 4 (Del A) | 315 | Ser AGC - Thr ACC |
| **Table S2:** Mutational profiles of 99 multidrug resistant isolates. (*Continued*) | | | | | | |
| 2788 | 516 | Asp GAC - Tyr TAC | -- | none | 315 | Ser AGC - Thr ACC |
| 2791 | 531 | Ser TCG - Leu TTG | -- | none | 315 | Ser AGC - Thr ACC |
| 294 | 526 | His CAC - Tyr TAC | -- | none | 315 | Ser AGC - Thr ACC |
| 310 | 526 | His CAC - Tyr TAC | -- | none | 439 | Ins G |
| 314 | 531 | Ser TCG - Leu TTG | -- | none | 315 | Ser AGC - Thr ACC |
| 319 | 533 | Leu CTG - Pro CCG | 4 | 4 (Del A) | 315 | Ser AGC - Thr ACC |
| 32 | 526 | His CAC - Asp GAC | -- | none | 315 | Ser AGC - Thr ACC |
| 379 | 531 | Ser TCG - Trp TGG | -- | none | 315 | Ser AGC - Thr ACC |
| 415 | 526 | His CAC - Asp GAC | -- | none | -- | none |
| 433 | 531 | Ser TCG - Leu TTG | 4 + 115 | 4 (Del A) + 115 (Ins T) | -- | none |
| 480 | 526 | His CAC - Asn AAC | 4 | 4 (Del A) | 315 | Ser AGC - Thr ACC |
| 491 | 516 | Asp GAC - Tyr TAC | 4 | 4 (Del A) | 315 | Ser AGC - Thr ACA (2 nucleotides) |
| 529 | 526 | His CAC - Tyr TAC | 4 | 4 (Del A) | 315 | AGC - Thr ACC |
| 543 | 531 | Ser TCG - Leu TTG | 4 | 4 (Del A) | 315 + 399 | 315 (Ser AGC - Thr ACC) + 399 (Glu GAA - Glu GAG) |
| 563 | 531 | Ser TCG - Leu TTG | -- | none | 315 | Ser AGC - Thr ACC |
| 605 | 531 | Ser TCG - Leu TTG | -- | none | 463 | Arg CGG - Leu CTG |
|  | 545 | Leu CTG - Pro CCG |  |  |  |  |
| 640 | 531 | Ser TCG - Leu TTG | 4 | 4 (Del A) | 315 | Ser AGC - Thr ACC |
| 682 | 531 | Ser TCG - Leu TTG | -- | none | 315 | Ser AGC - Thr ACC |
| 711 | 531 | Ser TCG - Leu TTG | -- | none | 315 | Ser AGC - Thr ACC |
| 720 | 531 | Ser TCG - Trp TGG | -- | none | 315 | Ser AGC - Thr ACC |
| 78 | 531 | Ser TCG - Leu TTG | -- | none | -- | none |
| 790 | 545 | Leu CTG - Pro CCG | 1 + 4 | 1 (Val GTG - Ala GCG) + 4 (Del A) | -- | none |
| 826 | 531 | Ser TCG - Leu TTG | -- | none | 315 | Ser AGC - Thr ACC |
| 836 | 531 | Ser TCG - Leu TTG | -- | none | 315 | Ser AGC - Thr ACC |
| 837 | 526 | His CAC - Asn AAC | 4 | 4 (Del A) | 315 | Ser AGC - Thr ACC |
| 847 | 511 | Leu CTG - Pro CCG | -- | none | 315 | Ser AGC - Thr ACC |
|  | 516 | Asp GAC - Tyr TAC |  |  |  |  |
| 850 | 516 | Asp GAC - Tyr TAC | 4 | 4 (Del A) | 315 | Ser AGC - Thr ACC |
| 865 | 531 | Ser TCG - Leu TTG | -- | none | 315 + 485 | 315 (Ser AGC - Thr ACC) + 485 (Del G) |
| 884 | 526 | His CAC - Tyr TAC | 93 | Ala GCC - Thr ACC | -- | none |
| 885 | -- | none | -- | none | 315 | Ser AGC - Asn AAC |
| 886 | 508 | Thr ACC - Pro CCC | -- | none | 315 | Ser AGC - Thr ACC |
|  | 526 | His CAC - Tyr TAC |  |  |  |  |
| 89 | 511 | Leu CTG - Pro CCG | -- | none | 315 | Ser AGC - Thr ACC |
|  | 531 | Ser TCG - Trp TGG |  |  |  |  |
| 933 | 531 | Ser TCG - Leu TTG | -- | none | 315 | Ser AGC - Thr ACC |
| 96 | 516 | Asp GAC - Val GTC | -- | none | 315 | Ser AGC - Thr ACC |
| 972 | 526 | His CAC - Arg CGC | -- | none | 315 | Ser AGC - Thr ACC |
|  | 511 | Leu CTG - Pro CCG |  |  |  |  |
| 976 | 531 | Ser TCG - Leu TTG | -- | none | 315 | Ser AGC - Thr ACC |
| 981 | 531 | Ser TCG - Leu TTG | 4 | 4 (Del A) | 315 | Ser AGC - Thr ACC |
| 452 | -- | none | -- | none | -- | none |
| 358 | 526 | His CAC - Cys TGC | -- | none | 315 | Ser AGC - Asn AAC |
| 378 | 526 | His CAC - Asp GAC | -- | none | 315 | Ser AGC - Thr ACC |
| 02 | -- | none | -- | none | 315 | Ser AGC - Thr ACC |
| 155 | -- | none | -- | none | 315 | Ser AGC - Thr ACC |
| 150 | 526 | His CAC - Asp GAC | -- | none | 315 | Ser AGC - Thr ACC |
| 231 | 526 | His CAC - Asp GAC | -- | none | 315 | Ser AGC - Thr ACC |
| 285 | -- | none | -- | none | 315 | Ser AGC - Thr ACC |
| 107 | -- | none | -- | none | 315 | Ser AGC - Thr ACC |
| 03 | 516 | Asp GAC - Tyr TAC | 4 | 4 (Del A) | 315 | Ser AGC - Thr ACC |
| 07 | 471 | Met ATG - Ile ATT | -- | none | 315 | Ser AGC - ILe ATC |
|  | 531 | Ser TCG - Leu TTG |  |  |  |  |
| 11 | 531 | Ser TCG - Leu TTG | -- | none | -- | none |
| **Table S2:** Mutational profiles of 99 multidrug resistant isolates. (*Continued*) | | | | | | |
| 76 | 526 | His CAC - Tyr TAC | -- | none | 315 | Ser AGC - Thr ACC |
| 261 | 526 | His CAC - Asp GAC | -- | none | 315 | Ser AGC - Thr ACC |
| 88 | 531 | Ser TCG - Leu TTG | -- | none | 315 | Ser AGC - Thr ACC |
