# Supplementary figures and images for: Multidrug Resistant Mycobacterium tuberculosis: A Retrospective katG and rpoB Mutation Profile Analysis in Isolates from a Reference Center in Brazil
Source: PLoS One. 2014 Aug 5;9(8):e104100. doi: 10.1371/journal.pone.0104100 (PMC4122415; doi:10.1371/journal.pone.0104100)

**Table S4**: Cluster Associations between *IS6110* - RFLP and Spoligotyping data.


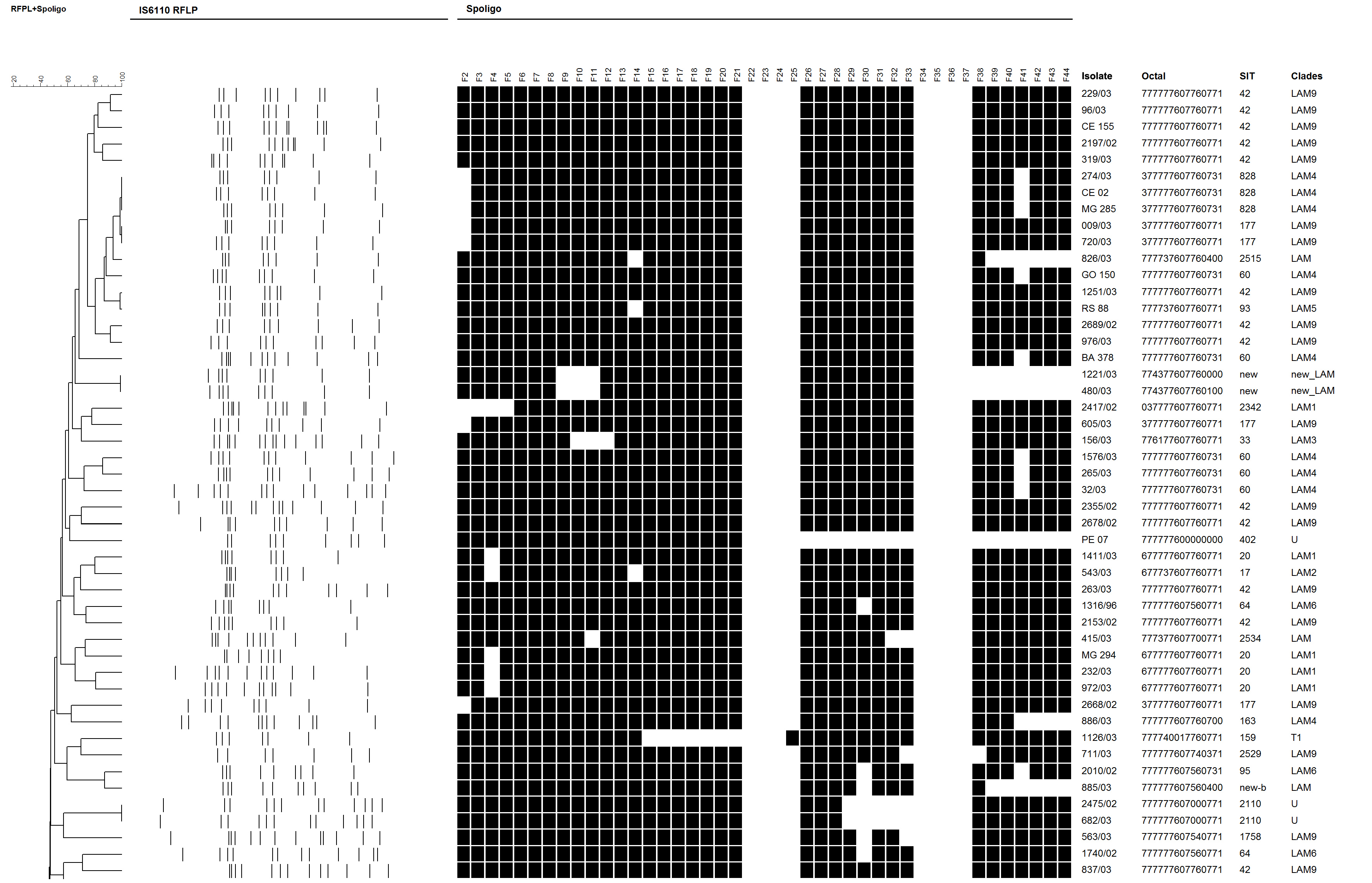


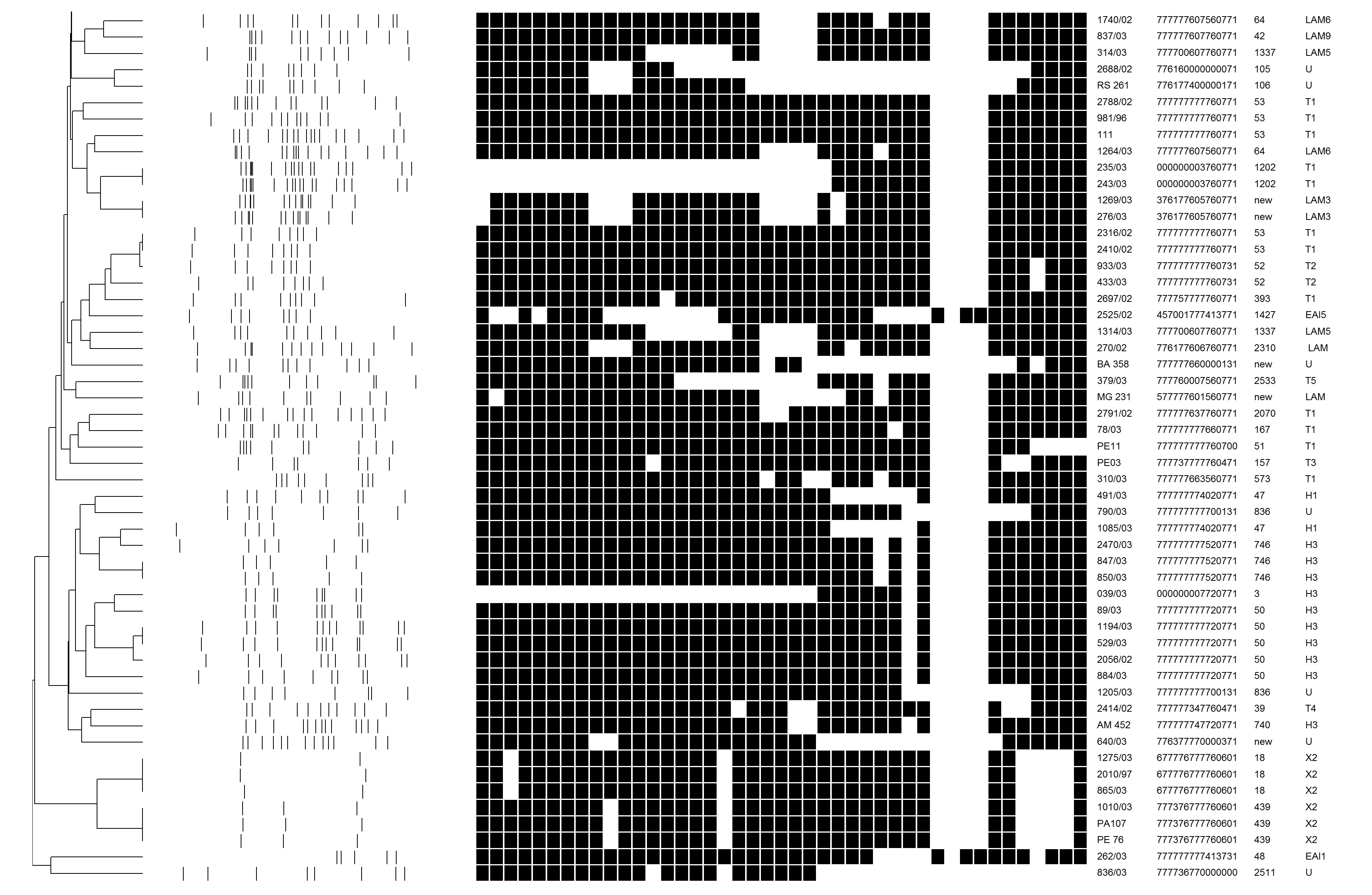


U = Unknow

Supplement: Table S4 — Table with cluster associations between IS6110 - RFLP and Spoligotyping data. (DOC) [file pone.0104100.s004.doc]
