# Supplementary material for: Multidrug Resistant Mycobacterium tuberculosis: A Retrospective katG and rpoB Mutation Profile Analysis in Isolates from a Reference Center in Brazil
Source: PLoS One. 2014 Aug 5;9(8):e104100. doi: 10.1371/journal.pone.0104100 (PMC4122415; doi:10.1371/journal.pone.0104100)
Supplement: Figure S1 — Map of Brazil showing the different states and the number of isolates analyzed. (PDF) [file pone.0104100.s005.pdf]

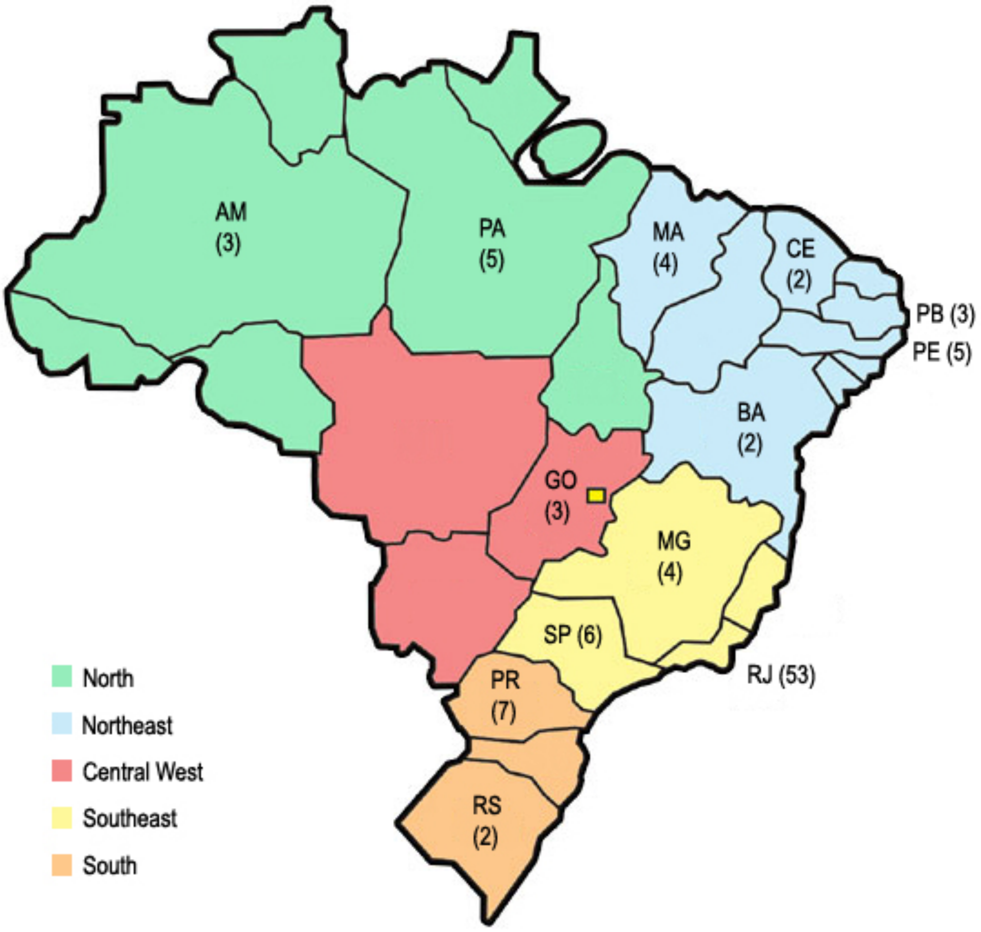

Figure S1: Map of Brazil showing the different states and the number of isolates analyzed. States: AM – Amazonas; PA – Pará; MA – Maranhão; CE – Ceará; PB – Paraíba; PE – Pernambuco; BA – Bahia; GO – Goiás; MG – Minas Gerais; RJ – Rio de Janeiro; SP – São Paulo; PR – Paraná; RS – Rio Grande do Sul.
